# Current Understanding of Flavonoids in Cancer Therapy and Prevention

**DOI:** 10.3390/metabo13040481

**Published:** 2023-03-27

**Authors:** Mohd Farhan, Asim Rizvi, Mohammad Aatif, Aamir Ahmad

**Affiliations:** 1 Department of Basic Sciences, Preparatory Year Deanship, King Faisal University, Al Ahsa 31982, Saudi Arabia; 2 Department of Kulliyat, Faculty of Unani Medicine, Aligarh Muslim University, Aligarh 202002, India; 3 Department of Public Health, College of Applied Medical Sciences, King Faisal University, Al Ahsa 31982, Saudi Arabia; 4 Translational Research Institute, Academic Health System, Hamad Medical Corporation, Doha P.O. Box 3050, Qatar

**Keywords:** flavonoids, polyphenols, anticancer, apoptosis, molecular mechanisms

## Abstract

Cancer is a major cause of death worldwide, with multiple pathophysiological manifestations. In particular, genetic abnormalities, inflammation, bad eating habits, radiation exposure, work stress, and toxin consumption have been linked to cancer disease development and progression. Recently, natural bioactive chemicals known as polyphenols found in plants were shown to have anticancer capabilities, destroying altered or malignant cells without harming normal cells. Flavonoids have demonstrated antioxidant, antiviral, anticancer, and anti-inflammatory effects. Flavonoid type, bioavailability, and possible method of action determine these biological actions. These low-cost pharmaceutical components have significant biological activities and are beneficial for several chronic disorders, including cancer. Recent research has focused primarily on isolating, synthesizing, and studying the effects of flavonoids on human health. Here we have attempted to summarize our current knowledge of flavonoids, focusing on their mode of action to better understand their effects on cancer.

## 1. Introduction

A number of studies have pointed to the importance of a plant-based diet in warding off conditions that can lead to cancer [1]. Vegetables contain a number of bioactive components, including phenolic compounds, carotenoids, and, most notably, flavonoids, which may contribute to the plant-based diet’s health advantages. The amount of research focused on exploring flavonoids thoroughly has risen significantly in the most recent years as a result of these potential uses.

**Figure 1:**
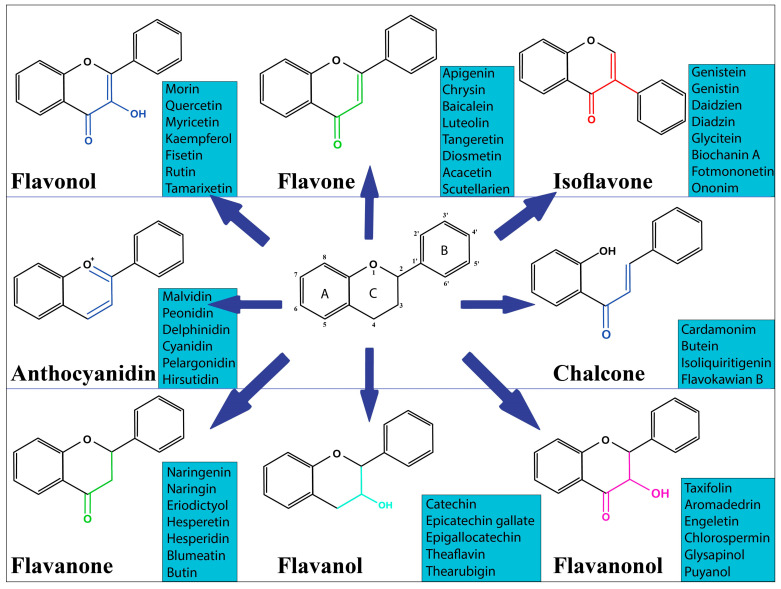
General structure of the flavonoid (center) and its subclasses.

As a class of phenolic chemicals generated by plants, flavonoids are classified as secondary metabolites. They can be found in a wide variety of photosynthetic species and are particularly prevalent in meals and beverages derived from plants, though their precise makeup varies greatly. Two benzene rings (A and B) are joined to the heterocyclic pyranic ring (C) to form the chemical structure’s 15-carbon skeleton [2]. There are many different types of flavonoids, and they may be broken down into several different classes: anthocyanins, flavones, flavonols, chalcones, isoflavones, flavanones, flavanonols, and flavanols [3]. This difference is due to the degree of unsaturation of the flavone ring and the oxidation of the carbonaceous ring (Figure 1), the key skeletons of the flavonoid.

It is challenging to establish epidemiologic correlations regarding the impact of flavonoids on human health versus disease due to the difficulty of estimating dietary intake due to the large quantitative and qualitative heterogeneity of flavonoids in a variety of vegetables and fruits. Flavonoids have been a part of the human diet in almost all geographic regions [4,5,6,7,8,9,10,11,12,13,14,15,16,17,18,19,20]. Details of the geographical consumption of flavonoids are beyond the scope of this review.

### 1.1. Aim of the Study

The review focuses on the anticancer characteristics of flavonoids by focusing on inflammation, migration of cancer cells, invasiveness, and metastasis development, which are crucial processes in the course of cancer. While it has been established that flavonoid consumption reduces the risk of cancer [21,22,23,24,25,26], this study aims to present a current assessment in relation to the connection between dietary flavonoids and their use in chemotherapy and chemoprevention, focusing on a targeted and individualized anticancer approach.

### 1.2. Source of the Data

After searching the PubMed bibliographic database using the terms “cancer” and “flavonoids” or “flavanones” or “flavonols” or “flavones” or “flavanols” or “isoflavonoids” or “chalcones” or “anthocyanidins”, we were able to retrieve data from the available literature. In the section that is dedicated to the anticancer effects of flavonoids, we place emphasis on the most current scientific studies.

## 2. Overview of Dietary Flavonoids

Flavonoids are widely distributed in foods and beverages of plant origin, such as fruits, vegetables, tea, cocoa and wine. A large amount of literature exists regarding flavonoid content in foodstuffs, which is summarized below (Table 1).

A number of studies have summarized the vast body of research on the bioavailability and absorption of flavonoids [37,38,39]. The molecular weight, glycosylation, and esterification of flavonoids, among other things, can impact their bioavailability, leading to some doubt about their actual levels of bioavailability as well as absorption in the human body [39]. Other studies also provide an in-depth account of how flavonoids are metabolized after ingestion [38,40]. The largest levels of flavonoids are found in fruits and vegetables [41]. Flavonoids are found in a variety of foodstuffs and beverages, although they are most prevalent in fruits and vegetables. Depending on the kind of fruit, the primary subclasses of flavonoids vary: anthocyanins prevail in berries, whereas flavanols predominate in pome fruits, tropical fruits, and drupes.

Some cereals (barley, buckwheat, and common wheat) have average flavonoid levels. However, it is vital to remember that whole grains contain the highest quantities when raw, and levels are dramatically reduced when grains are treated with heat or refined for further use [42,43]. Flavonoid-rich foods include cocoa and its derivatives. Flavanols are the most abundant flavonoids in these foods, with cocoa containing the maximum amount of flavanols [44,45]. Tea infusions, notably black and green tea, have the highest quantities of flavonoids in non-alcoholic beverages, primarily flavanols [46,47,48]. Fruit juices, in particular apple juice, orange juice, grapefruit juice, and lemon juice, are the second most flavonoid-rich beverages [49]. Flavanones are the primary flavonoids found in citrus and grapefruit juices [50,51]. Food stuff and drinks rich in flavonoids (Table 2) are summarized below [52].

Besides influencing mammalian metabolism, flavonoids have been linked to a wide range of anti-inflammatory, antiviral, antiproliferative, and anticarcinogenic properties [53]. Certain pathological illnesses, such as gastric and duodenal ulcers, allergies, vascular fragility, and viral and bacterial infections, have gained a lot of attention because of the beneficial effects flavonoids have as antioxidants in preventing human diseases, including cancer and cardiovascular disease [54]. In general, flavonoids have been discovered to exhibit a variety of pharmacological properties [53]; these include antioxidant, antiallergic, antiinflammatory, antidiabetic, hepato- and gastro-protective, antiviral, and antineoplastic activity.

## 3. Effects of Flavonoids in Chemoprevention and Chemotherapy

**Figure 2:**
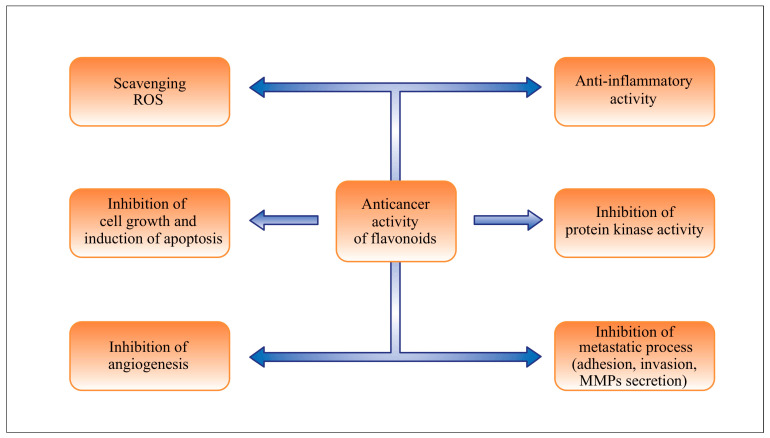
Flavonoids’ possible role in preventing cancer.

Flavonoids are effective anti-inflammatories as well as potent antioxidants that combat free radicals, which are related in a crucial way to many degenerative chronic diseases and are responsible for a wide range of biological processes (Figure 2). An increase in free radicals under pathological conditions not only causes cellular aging and death but also promotes carcinogenesis by causing damage to various molecule types, including nucleic acids, proteins, and lipids [4].

### 3.1. Role of Flavonoids in Inflammation

Inflammation constantly occurs in the body, and this is thought to be a contributing factor in cancer [55]. Carcinogenesis is a result of inflammation in a number of disorders. It is thought that *Clonorchis sinensis* infection is responsible for the chronic inflammatory infiltrate seen in cholangiocarcinoma of the biliary system [56]. Adenocarcinoma and lymphoma of the gastric mucosa-associated lymphoid tissue are both linked to Helicobacter pylori [57]. The incidence of hepatocellular carcinoma ranks as the third leading cause of mortality from cancer [58], and chronic hepatitis B and C virus infection is a risk factor. Human papillomavirus infection is a major contributor to human penile and anogenital cancer. In addition to this, there is evidence that schistosomiasis is linked to an increased risk of bladder cancer, and human herpesvirus type 8 infection has been associated with an increased risk of Kaposi’s sarcoma [59]. Carcinogenesis can also be influenced by persistent inflammation that is not caused by microorganisms [60]. Barrett’s metaplasia, esophagitis, and chronic pancreatitis are all examples of inflammatory disorders that have been linked to an increased risk of cancers of the pancreas, esophagus, and gallbladder [61,62]. Marjolin ulcers and skin cancer have also been associated with increased cancer probability [63]. Asbestos and mesothelioma [64], tobacco and bronchial cancer [64], persistent asthma and lung cancer [65], squamous cell carcinoma and ulcerative lichen planus [66], inflammation of the foreskin (phimosis) and penile cancer [67], and inflammation of the pelvis and ovaries and ovarian cancer [68] have all been linked. Chronic prostatitis, whether due to a bacterial infection or other non-infectious reasons, has been linked to the development of prostate cancer [69]. The data linking chronic inflammation to cancer is growing, suggesting a causal relationship between the two.

We have previously shown that Nrf2 is a transcription factor that can be possibly influenced by polyphenols to combat oxidative stress in malignancies [70]. Nrf2 is a nuclear factor erythroid 2-related factor 2 signaling pathway, which was shown to be activated in Swiss albino mice when they were exposed to the mutagen benzopyrene, which is commonly found in the smoke of cigarettes and the exhaust of automobiles [71]. The anti-inflammatory chrysin is a kind of flavonoid aglycone. By inhibiting the inositol-requiring enzyme-1/thioredoxin-interaction protein/nucleotide-binding oligomerization domain-like receptor protein 3 pathway, chrysin prevented lung damage from developing in LPS (lipopolysaccharide)-challenged mice [72]. In rats, it activated endothelial nitric oxide synthase and Nrf2 target genes such as SOD (superoxide dismutase) and catalase [73], which protected myocardial problems from hypercholesterolemia-triggered oxidative stress. Chrysin also greatly suppressed proliferation and promoted apoptosis in human cervical cancer cells [74] and colorectal cancer cells [75] via regulating several apoptotic genes and genes involved in the AKT/MAPK pathway. In light of these findings, we can identify two separate pathways by which flavonoids influence their impacts on inflammation and cell proliferation: they work on cell proliferation via modifying genes implicated in apoptosis and the AKT/MAPK (protein kinase B/mitogen-activated protein kinase) pathway and on inflammation by activating the Nrf2 pathway to suppress NF-kB and causing an anti-inflammatory action.

### 3.2. Relationship between Flavonoids and Oxidative Stress

**Figure 3:**
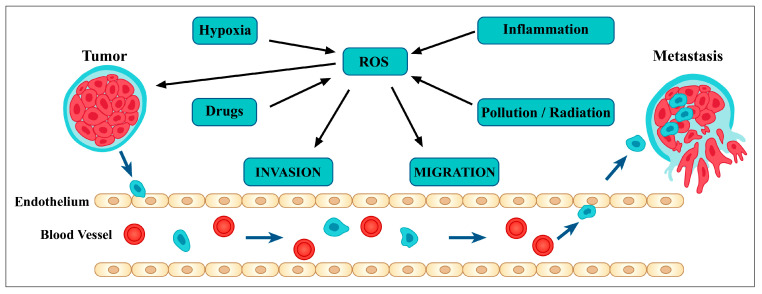
The role of oxidative stress in cancer development.

Due to an ineffective antioxidant mechanism, cancer cells are shown to possess a higher concentration of reactive oxygen species (ROS), primarily hydrogen peroxide, within their cytoplasm than normal cells. When the glutathione (GSH/GSSG) ratio is right, hydrogen peroxide is neutralized and converted to water in healthy cells [76]. Reduced glutathione levels cause hydrogen peroxide to undergo a chemical reaction that results in the extremely reactive hydroxyl radical (OH) [76], which in turn damages DNA and causes mutations in several genes, including tumor suppressor genes. This is the first important step that sets off the cascade of events that culminates in carcinogenesis [77]. Cancer has at least three distinct phases: inception, promotion, and advancement. All of these steps involve oxidative stress in some way (Figure 3). ROS can cause mutations in genes and structural changes to DNA during the beginning phase. By altering gene expression, intercellular communication, and intracellular signaling pathways, ROS play a crucial role in promoting either cell growth or death [77,78]. Last but not least, oxidative stress promotes tumor growth by causing further mutagenesis in the incipient cell population [79]. Many anticancer medications work to induce apoptosis by increasing the ROS levels already present in tumor cells [80]. Flavonoids are commonly thought of as antioxidants, but they can also have pro-oxidant action and induce death in cancer cells.

Grapefruit, orange, lemon, and lime peel are particularly rich sources of the flavanone naringenin. It inhibited cell proliferation and triggered programmed cell death in a variety of human tumor cells [81,82]. It also reduced gastric cancer and hepatocellular carcinoma cell invasiveness and metastasis [83,84]. The pro-oxidant action of naringenin was due to the suppression of glutathione-reductase, glutathione-S-transferase, and glyoxalase activities in tumor cells [85]. This, in turn, allowed for the buildup and the augmentation of lipid peroxidation, leading to cell membrane damage. Intriguingly, the safety and pharmacokinetics of naringenin [86] have recently been emphasized by a phase I clinical investigation. A concentration of 43 µM of naringenin was found in plasma 4 h after a single dosage of Citrus sinensis extract (i.e., sweet-orange) was administered. Iron, one of the key metal elements, maintains regular physiological activity in the human body, which is mostly engaged in important physiological processes such as oxygen transport, electron transport, DNA synthesis, and many enzymatic reactions. Iron is absorbed through the gastrointestinal tract, transported into the bloodstream, and disseminated throughout the body, and there are no natural routes of excretion following absorption [87]. When there is too much iron in the body, excess iron can be accumulated in tissues and organs, generate lipid peroxidation, and effects cell damage, which can lead to cancer, hematological illnesses, and other chronic and regularly encountered diseases. Research on iron excess largely focuses on liver fibrosis, liver cancer [88], and hematological illnesses [89]. Deferrioxamine, deferiprone, and deferasirox are commonly used to treat iron overload as iron chelators; however, they are prone to side effects. Ferroptosis, as programmed apoptosis, is characterized by excessive accumulation of lipid peroxides and reactive oxygen species [90]. Ferroptosis, typically combined with iron overload, produces tissue damage principally driven by an excess of iron and lipid peroxidation. At the same time, ferroptosis induces ferritin degradation, impairs iron metabolism and leads to iron overlaod [91]. The occurrence of ferroptosis leads to normal tissue and organ damage and loss of function, which is directly involved in the onset, development and prognosis of various chronic and common diseases [92]. Flavonoids, which have the capacity to suppress reactive oxygen species, scavenge free radicals and regulate iron homeostasis, are less expensive and have fewer side effects, and are promising novel iron chelators [93]. Our own research indicated that EGCG is capable of killing chemically induced hepatocellular carcinoma cells in rats. It was shown that this was accompanied by perturbations in the oxidative stress response of these cells [94].

### 3.3. The Role of Flavonoids in Apoptosis and Autophagy

Inducing cancer cell apoptosis is a current focus of the quest for anticancer treatments [95]. Cell death can be prevented by activating the apoptotic cascade; however, cancer cells are able to evade this defense. Inducing medication resistance also facilitates tumor growth [95]. Casticin is a flavonoid that is derived from the Vitex agnus-castus species and used as an anti-inflammatory in Chinese medication. Casticin is capable of activating apoptosis through the regulation of Bcl-2 and other pro-survival proteins [96]. This compound has been shown to trigger the intrinsic route of apoptosis in a large number of cancer cell lines derived from a wide variety of cancers. This is accomplished by downregulating Bcl-2, Bcl-xL, and survivin while simultaneously upregulating Bax [96]. Vitexin, an extract of the Chinese herb known as Crataegus pinnatifida, is a flavonoid that has been demonstrated to kill cancer cells by decreasing the Bcl-2/Bax ratio, mitochondrial cytochrome c release, and caspase-3 cleavage in human non-small cell lung cancer A549 cells [97].

One of the most common flavonoids found in onions and broccoli is called quercetin. This flavonoid was able to inhibit the progression of a line of human metastatic ovarian cancer called PA-1 [98]. It did this by increasing the expression of pro-apoptotic molecules such as caspase-3 and caspase-9 and decreasing the expression of anti-apoptotic molecules such as Bcl-2 and Bcl-xL [98].

Autophagy is an ancient catabolic mechanism that controls cell death in a favorable way when triggered by stress. Several cancer treatments induced autophagy, suggesting that this process could be used as a therapeutic method [99]. Allspice has a wide variety of flavonoids that are concentrated in its water extract. Cell death and autophagy were triggered in breast cancer cells by inhibiting the Akt/mammalian target of the rapamycin (mTOR) pathway [100]. Similarly, kaempferol downregulated CDK1/cyclin B and triggered G2/M arrest via inducing autophagy in SK-HEP-1 (human hepatic cancer cells) [101]. This was achieved via Akt signaling and adenosine monophosphate-activated protein kinase (AMPK). In addition, the isoflavone genistein’s anti-tumor action may stem from the fact that it induces autophagy in various cancers, including those of the breast, prostate, and uterine [102].

It has been demonstrated that the flavonoid wogonin induces the death of cancer cells by inhibiting autophagy [103]. In contrast, it has been demonstrated that several flavonoids trigger cell death via autophagy. Curcumin, for instance, can trigger autophagy and death in chronic myeloid leukemia cells by downregulating the Bcl-2 protein [104]. Quercetin causes widespread autophagy in epithelial cancer cells, resulting in cell cycle arrest and apoptosis [105], etc. Based on findings, newly discovered flavonoids, including IH, or isorhamnetin, GN, or genkwanin, and Aca, or acacetin, may be autophagic inducers that promote the accumulation of autophagosomes in breast cancer cells [106]. Initially, treatment of breast cancer cells with these flavonoids led to an increase in the production of EGFP-LC3 puncta and the accumulation of LC3B-II [106]. Secondly, treatment with these flavonoids reduced the levels of p62, a protein that functions as a cargo receptor for the autophagic destruction of substrates and is a sign of autophagy induction [106]. Thirdly, treatment with these flavonoids enhanced the levels of ATG5, a molecule regarded as important for the induction of autophagy [106]. By evaluating the colocalization of EGFP-LC3 and mRFP-LC3 puncta, it was determined that exposure to these flavonoids led to the creation of a high number of red-only puncta, comparable to that observed in cells treated with rapamycin, a common autophagy inducer. The data indicated that ATG5 contributes to the autophagosome accumulation produced by flavonoids.

Our recent study also highlighted that EGCG and its structurally related catechins are capable of inducing apoptosis and autophagy via signaling pathways in various cancer models [107].

### 3.4. Relationship between Flavonoids and Cancer Stem Cells

Self-renewing and capable of both launching and maintaining tumor growth, cancer stem cells (CSCs) make up a minute but a crucial fraction of the tumor. In addition to being essential for the development of the disease, CSCs in cancer are also involved in its maintenance, progression, and metastasis [108]. Emerging research suggests that flavonoids and other dietary phytochemicals can act as effective agents against CSCs [109]. It has been shown, for instance, that naringenin, similar to hesperidin [110], suppresses breast cancer stem cells by elevating p53 and the estrogen receptor.

The flavone known as apigenin can be found in the highest concentrations in chamomile, celery, and parsley. It is believed that the anticancer properties of apigenin are effective in treating the brain tumor known as glioblastoma [111]. Kim et al. [111] found that apigenin and quercetin were able to diminish the self-renewal ability and invasiveness of glioblastoma stem-like cells by altering the c-Met signaling pathway. This was accomplished by inhibiting the growth of cancerous tumors. Apigenin lowers the stem-cell-like features and tumorigenic potential of triple-negative breast cancer cells [112]. Additionally, apigenin enhances the antineoplastic activity of cisplatin in CD44+ prostate cancer stem cell populations [113]. It was discovered that the flavone luteolin [114], which is found in a wide variety of foods (including celery, carrots, peppers, and olive oil), could reduce the ability of oral cancer stem cells to self-renew and could restore their sensitivity to radiotherapy. With the ability to inhibit the growth of cancer cells, flavonol quercetin has attracted the attention of researchers in the medical field [115]. It has been demonstrated that quercetin can precisely target CSCs in a variety of tissues. These include the pancreas [116], the breast [117], and the pancreas [118].

### 3.5. Flavonoids Inhibit Angiogenesis and Metastasis

Inhibiting angiogenesis is a fascinating function of flavonoids. The process of angiogenesis, which entails the formation of new blood vessels, is essential for normal tissue expansion, wound healing, as well as embryonic development; however, it is a deleterious feature when a tumor is concerned because it provides cancer cells with a richer environment in which to thrive and proliferate [119]. Cancer, angiogenesis, and inflammation are all interconnected phenomena because they are all tightly regulated by numerous inducers, such as vascular endothelial growth factor (VEGF) and adhesion molecules, and numerous inhibitors, such as angiostatin and thrombospondin [119]. Angiogenesis is a critical step in cancer progression, invasion, and metastasis, making the development of inhibitors to stop it a focus of anticancer research in recent years. In response, the Food and Drug Administration (FDA) authorized the use of a plethora of anti-angiogenesis medications in cancer therapy [120,121]. Research is being done to see whether there are any new chemicals that can prevent tumors from re-growing blood vessels. It has been shown that the O-methylated flavone wigonin, a flavonoid-like chemical molecule synthesized by *Scutellaria baicalensis*, suppresses LPS-induced angiogenesis both in vitro and in vivo [121]. Modulating the expression of (VEGF), metalloproteases (MMP), and epidermal growth factor receptor (EGFR) [122], is how genistein suppresses angiogenesis. Kaempferol acts on VEGF receptor 2 to suppress angiogenesis in VEGF-stimulated human umbilical vein endothelial cells (HUVECs). P13kt/Akt, the mitogen-activated protein kinase (MEK), and the extracellular signal-regulated kinase (ERK) pathways all play a role in facilitating this process [123].

By inhibiting the expression of MMP-9 metalloproteinase and interleukin-8 (IL-8) [124], the glycosyl dietary flavonoid luteolin (8-C-d-glucopyranoside) inhibits tumor invasion in 12-O-tetradecanoylphorbol-13-acetate (TPA)-treated MCF-7 breast cancer cells. Quercetin inhibited the metastasis of gastric cancer cells by inhibiting urokinase plasminogen activator (uPA)/uPA receptor (uPAR) function through the regulation of nuclear factor kappa B (NF-kB), protein kinase C (PKC), extracellular signal-regulated kinases 2 and 3, and AMPK [125]. Yao et al. [126] discovered that luteolin induces dose-dependent death in A375 human melanoma cells, where it blocks proliferation, migration, and invasion. As a result, Akt and PI3K phosphorylation were both suppressed in this model cell system. Similar authors have compiled experimental evidence showing that luteolin suppresses the expression of MMP-2 and MMP-9 while allowing the overexpression of tissue inhibitors of metalloproteinase (TIMP)-1 and TIMP-2 [126]. In addition, experimental data showed that luteolin dramatically suppressed tumor growth of A375 cells in a mouse xenograft model, demonstrating that the anticancer activity is derived from the down-regulation of MMP-2 and MMP-9 expression via the PI3K/Akt pathway [126].

Although quercetin has been shown to have anti-proliferative properties in a number of cancer cell lines, it may not have much of a place in cancer treatment on its own. Quercetin was found to have anticancer activity in vitro under hypoxia but to have a much less effect against 4T1 breast cancer under normoxia [127]. Surprisingly, in vitro and in vivo, quercetin combined with doxorubicin (DOX) demonstrated preferential action against 4T1 cells. The concentration of quercetin used in the in vitro studies was determined by its ability to significantly downregulate HIF-1 and its ability to inhibit growth less in cancer cells in the presence of hypoxia; the dose of quercetin used in the in vivo studies was based on the optimal dose that resulted in the greatest antitumor effect as a single agent. The research showed that quercetin improves the therapeutic index of DOX by increasing its cytotoxicity against tumor cells while protecting normal cells from DOX-induced damage in vitro and in vivo [127].

### 3.6. The Role of Flavonoids in Cancer Cell Differentiation

The goal of differentiation therapy is to slow the growth of cancer cells through the induction of differentiation [126]. As opposed to traditional chemotherapy, differentiation therapy has the benefit of being less toxic and, thus, resulting in fewer adverse effects for the patient [128]. Transglutaminase type 2 is involved in the differentiation induced by quercetin and pelargonidin malignant B16-F10 murine melanoma cells [129]. Differentiation therapy, including all-trans retinoic acid (ATRA), is commonly used for patients suffering from acute-promyelocytic leukemia (also known as APL). However, medication resistance develops over time with continued treatment, necessitating ever-increasing dosages [130]. In order to combat the phenomenon of drug resistance, new medicines with higher differentiation induction activity need to be developed. In this regard, flavonoids possess several really fascinating features. Flavanoids can cause APL cells to differentiate. The structure of flavones, however, may be critical for inducing cell differentiation. Quercetin, apigenin, and luteolin all cause granulocyte differentiation in APL cells, while echinacea induces monocyte differentiation. However, galangin, kaempferol, and naringenin failed to promote the differentiation of APL cells [130].

Epigallocatechin-3-gallate (EGCG), a green tea polyphenol, has recently been discovered to produce effects that are comparable to those of ATRA on the differentiation of APL HL-60 and NB4 cells, as reported by Moradzadeh et al. [131] EGCG inhibited the expression of histone deacetylase 1 in both cell lines and also inhibited the expression of the clinically important marker PML-RAR in NB4 cells. In the K562 cell line (chronic myeloid leukemia (CML)), wogonin stimulated cell differentiation. Imatinib-sensitive and resistant patient-derived primary CML showed the same trend. Moreover, GATA-1 was upregulated in these cells, and interaction between GATA-1 and FOG-1 (transcriptional coactivator) was enhanced [132]. Several findings provide credence to the idea that flavonoids could be used to treat people with various forms of cancer. Treatment with flavonoids has been shown to induce differentiation in malignant cells isolated from a variety of solid tumors [133]. The flavonoids genistein [102,122] and isoliquiritigenin [134] have been shown to stimulate cell differentiation in breast cancer stem cells.

The flavonoid dihydromyricetin (DMY) was found to synergize with ATRA to induce cell differentiation in the process of treating APL NB4 cells [135]. It has been found that STAT1, which controls several myeloid transcription factors and proteins involved in the cell cycle, plays an essential function in the completion of the differentiation process that is prompted by ATRA in myeloid cells [135]. It was necessary for there to be an enhanced activation of the p38 MAPK/STAT1 signaling pathway in order for DMY and ATRA to have their synergistic effects on differentiation. Intriguingly, DMY alone failed to stimulate differentiation and suppressed p38 MAPK phosphorylation, leading to diminished STAT1 activity [135]. It follows that any and all flavonoids may serve as synergistic boosters of differentiation from standard medications.

### 3.7. Immunomodulatory Effect of Flavonoids

In recent years, there has been much debate about how to prevent, diagnose, predict, and treat cancer because the number of cases has been going up. Flavonoids have specific effects on the immune system that could be important for a number of cancers. In recent years, flavonoids have become widely known as natural polyphenol compounds that could be used to treat a number of diseases. This is because flavonoids can affect the immune system, fight free radicals, and kill cancer cells. However, it is still not clear how flavonoids affect the immune system and stop tumors from growing [136,137,138].

It is considered that the immunomodulatory action is one of the most important flavonoid anticancer mechanisms: (a) flavonoids augment the activity and cytotoxicity of NK cells toward tumor cells by upregulating their activating receptors, (b) Flavonoids boost the cytotoxicity of cytotoxic T lymphocytes (CTL cells) and decrease regulatory T cell (Treg cell) activity against tumor cells [139,140]. A recent study demonstrated that hesperetin can prevent the growth of tumors by enhancing the immunological response of CTL cells and decreasing the immune response of Treg cells [136]. Curcumin and apigenin appeared to promote CD8+ cell infiltration in mouse tumor tissue, according to another study [141]. (c) Flavonoids prevent the synthesis of numerous pro-inflammatory cytokines. The role of the inflammatory tumor microenvironment in the evolution of malignant tumors cannot be denied [142]. Nevertheless, flavonoids displayed anti-inflammatory activity by suppressing pro-inflammatory cytokine release, blocking the NF-kB and NLRP3 signaling pathway, reducing chemokine synthesis, and other mechanisms [143] (d) Flavonoids reduce the expression of PD-1 and/or PD-L1 on T cells. Baicalein and baicalin suppressed PD-L1 expression and increased the tumoricidal activity of TCL cells in vitro, according to a previous study [144]. In addition to inhibiting PD-L1 and p-STAT, apigenin inhibited PD-L1 and p-STAT in triple-negative mammary cancer cells [145].

The immunomodulatory effects of flavonoids in cancer have also been demonstrated by multiple cell and animal models via mechanistic studies [146,147,148,149]. Flavones exhibit immunomodulatory action at concentrations below those needed to destroy cancer cells [150,151]. The capacity of flavonoids to modulate inflammation, a characteristic of cancer, gives new chances to control immune cells in the tumor microenvironment (TME) [152]. TME is a dynamic ecosystem composed of an extracellular matrix (ECM), vascular networks, and multiple cell types, such as stromal cells, fibroblasts, myofibroblasts, adipose cells, and immune cells, among others. A favorable TME can promote the growth and metastasis of tumor cells [153,154,155,156]. It has been demonstrated that pathologically elevated inflammation in the TME is an etiologic factor in numerous forms of cancer. It has been demonstrated that the TME possesses dysregulated metabolic characteristics [157]. Therefore, nutrient-mediated modification of TME may provide a significant, side-effect-free therapy option. The metabolic dysregulation in the TME suppresses the immune response of natural killer (NK) cells [158]. Moreover, myeloid-derived suppressor cells (MDSCs), Treg cells, and tumor-associated macrophages (TAMs) can contribute to tumor immunoevasion [159,160]. The expansion of immunosuppressive cells in the TME has been associated with a poor prognosis in numerous malignancies [161,162,163,164]. In order to cure cancer, dietary therapies aimed at reducing the number of immunosuppressive cells or reactivating their anti-tumoricidal function have attracted considerable interest.

Dietary flavonoids can target the tumor microenvironment by reprograming Treg cells and TAMs and decreasing angiogenesis [165]. Vadimezan (DMXAA), a tiny flavonoid-like molecule, reprograms TAMs, leading to an increase in TNF production and the activation of CD8+ T cells [166,167]. Baicalin has been found to reprogram TAMs in a mouse model of hepatocellular carcinoma (HCC) by activating the NF-kB signaling pathway [168]. Luteolin, a common flavone, inhibited the transcription factor STAT6-dependent release of the chemokine CCL2, a critical regulator of the number of TAMs in the TME, and reduced the migration of Lewis lung carcinoma cells [169]. Grape antioxidants can also target NF-kB by decreasing its DNA-binding ability, hence preventing the invasion of cancer cells [170]. Dietary supplementation with grape seed rich in proanthocyanidins was found to reduce ultraviolet B (UVB 280–320 nm)-induced skin tumor development via the reduction in oxidative stress, activation of signaling pathways of mitogen-activated protein kinases and NF-kB, and immunosuppression via cytokine changes [171]. Thus, additional research is required to determine how dietary flavonoids decrease tumor growth and immune evasion, as well as human clinical trials.

### 3.8. Incorporating Flavonoids with Chemotherapy

The clinical effectiveness of existing anticancer medications can be increased through the use of combination therapies including numerous substances [85,172]. The need for novel approaches to enhance chemotherapy sensitivity while reducing unintended side effects persists in the face of multi-drug resistance and tumor recurrence. For this reason, flavonoids’ anticancer properties have made them attractive molecules. Arsenite and delphinidin (one of the anthocyanin compounds) have been shown to have antiproliferative effects in human NB4 and HL-60 APL cells in a study conducted by Yuan et al. [173]. Delphinidin modulated the amount of glutathione and decreased the activity of NF-kB, which resulted in arsenite-resistant leukemia cells becoming more susceptible to death. In addition to this, they showed that combination therapy was selectively effective, as it amplified the cytotoxic impact of arsenite on cancerous cells [173].

Additionally, flavonoids and other treatments had a positive impact on different cell types that had solid tumors stabilized. It has been shown that quercetin inhibits heat-shock protein 27 in order to make human glioblastoma U87 and U251 cells more sensitive to temozolomide (an oral alkylating chemotherapy medication) [174]. In glioblastoma U87 and T98G cells, the addition of the isoflavone biochanin A and the drug temozolomide affected the viability of the cells, raised the expression of the protein p-p53, and increased the expression of cell survival proteins (EGFR, p-Akt, p-ERK, membrane-type-MMP1, and c-myc) [175]. Cancer cells treated with a combination of drugs saw a major shift from anaerobic to aerobic forms of energy consumption, as well as a stop in the cell cycle at the G1 phase [124].

In ovarian cancer SK-OV-3 cells that were resistant to cisplatin, synergistic sensitization to cisplatin was achieved by co-treating the cells with morin and cisplatin. In addition, it has been hypothesized that the sensitization of ovarian cancer cells to cisplatin is accomplished by the downregulation of galectin-3 by morin [176], which is a protein that is crucial for a variety of cellular functions, including apoptosis. In addition, morin hydrate was able to reverse the acquired resistance of cisplatin-resistant hepatocellular carcinoma HepG2DR cells by inhibiting autophagy that was dependent on PARP-1/HMGB1 [177].

In chemoresistant colon cancer LS174 cells, the combination of kaempferol with 5-FU produced a synergistic inhibitory effect on cell viability, increased apoptosis, and triggered cell cycle arrest [178]. Additionally, kaempferol inhibited the generation of reactive oxygen species (ROS) and altered the expression of JAK/STAT3, MAPK, PI3K/AKT, and NF-kB signaling in these cells [178].

In addition, other groups of flavonoids, such as chalcones, have also been shown to demonstrate high chemosensitizing properties in cancer model systems [179]. In resistant colon cancer SW480 cells, the addition of xanthohumol, which is a prenylated flavonoid derived from hops, and the chemotherapeutic drug SN38, which is the active metabolite of irinotecan, resulted in a reduction in cell survival in comparison to the use of SN38 alone. Therefore, xanthohumol has the potential to be exploited as a chemosensitivity enhancer for SN38 [179]. In gemcitabine-resistant NSCLC cells, a further chalcone called flavokawain-B demonstrated powerful anti-cancer properties [180]. It did this by triggering apoptosis and ROS generation and disrupting the PI3K/Akt signaling pathway.

NF-kB controls the transcription of many genes involved in a wide variety of cellular functions and processes, including cell cycle progression, survival, protection against oxidative stress, invasion, and metastasis [181]. A rise in resistance to radiotherapy and chemotherapy is associated with the acquisition of all these characteristics, which are associated with activated NF-kB. The stimulation of manganese superoxide dismutase, a nuclear-encoded mitochondrial enzyme involved in the control of oxidative stress, is a particularly well-known involvement of NF-kB in the development of radiation resistance [182,183,184]. The up-regulation of various anti-apoptotic genes is another significant role played by NF-kB in facilitating adaptive resistance to radiation. Several investigations have shown that BCL-2 and BCL-xL have B sites in their respective promoters [185]. Radiotherapy success in numerous tumor types, including prostate, laryngeal, and head and neck malignancies, may be predicted by the expression of BCL-2, according to a retrospective analysis. These observations suggest that elevated BCL-2 expression is a key mechanism by which cancer cells evade the killing effects of ionizing radiation [186].

EGFR is one of the most explored molecular targets in non-small cell lung cancer (NSCLC), and tyrosine kinase inhibitors can cure advanced NSCLC. EGFR mutations and compensatory pathway activations may reduce the efficacy of tyrosine kinase inhibitors. Green tea’s major bioactive ingredient, epigallocatechin-3-gallate (EGCG), inhibits cancer cells from overexpressing EGFR [187]. Recently, EGCG was shown to block EGFR signaling activation in three NSCLC cell lines with wild-type or mutant EGFR [187]. Thereafter, proliferation, apoptosis, migration, and vinculin expression were examined. EGCG polyphenol suppresses NSCLC cell growth and migration in multiple ways. These findings may help evaluate EGCG as an NSCLC treatment adjuvant.

### 3.9. Flavonoids Nanoformulations in Cancer Therapy

**Figure 4:**
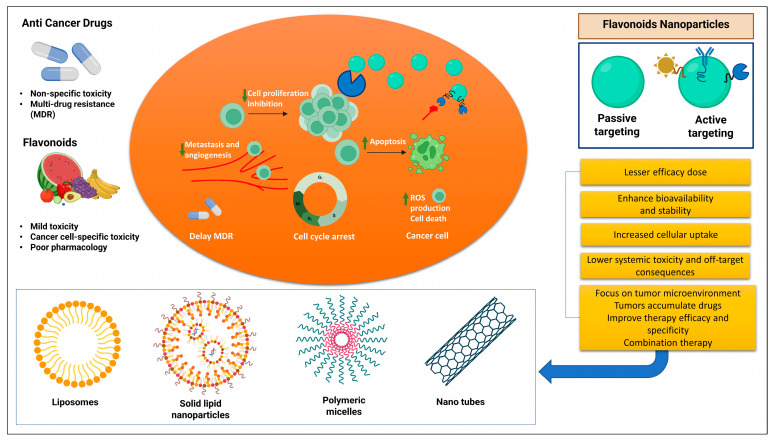
Nanoparticles loaded with flavonoids for use in cancer treatment.

In recent decades, significant attempts have been undertaken to successfully deliver medications to solid tumors. Even then, the cancer therapy effectiveness is limited [188,189]. Drug uptake, systemic circulation, and drug accumulation in tumor lesions are a few of the obstacles that impede the delivery of anticancer drugs to the target site in tumor cells [190]. The tumor microenvironment has a substantial impact on cancer diagnosis, treatment, and drug delivery [191]. As a result, altering the tumor microenvironment as a way to enhance the efficacy of cancer treatments is gaining increasing interest. By producing nanomedicines [192], anticancer drugs have been designed as high-precision and targeted therapeutics. In the past few years, scientists have tested a number of nanoparticles for application as carriers of flavonoids alone or in combination with chemotherapy in drug delivery in cancer treatment. There are organic nanoparticles, such as solid lipid nanoparticles, protein nanoparticles, and liposomes, and inorganic nanoparticles, such as metallic nanoparticles and silica nanoparticles [193,194,195]. When compared to conventional treatments, nanoparticles have a better absorption and preferential targeting of medicines to the tumor due to their size and unique cancer pathology (Figure 4). There are two primary approaches to this goal: “passive” and “active” targeting [193]. Cancer’s distinctive alterations to the vasculature make passive targeting a realistic option. Rapid tumor growth can cause the improper formation of blood arteries and junctions, which can lead to bleeding. Nanoparticle formulations are able to bypass these loose connections and accumulate preferentially at the tumor location because of their unusually small size. This is referred to as the EPR effect, or enhanced penetration and retention [193]. In the active targeting of nanoparticles, a targeting moiety is added to the nanoparticle system in order to target alterations in cancer cell biology that are greatly elevated in comparison to the healthy surrounding cells and tissues. Through ligand–receptor interactions, nanoparticles can identify and bind to their intended targets; once bound, the nanoparticles are taken up by the cell, and the drug is released inside the cell, with significantly less drug escaping into the surrounding environment than in passively targeted systems [193].

Flavonoids encapsulated in metal nanoparticles could be used to cure cancer. Activation of the caspase cascade via the Bcl-family proteins in the mitochondrial pathway has been shown to be effective in causing apoptosis (programmed cell death) in cancer cells [196]. EGCG-conjugated gold nanoparticles are one such example. Hsieh et al. [196] found that the addition of gold nanoparticles altered the biological activity of EGCG via affecting the quantity of free radicals. In addition, gold nanoparticles improve receptor-mediated endocytosis, a process by which the medicine is taken up specifically by bladder tumor cells. EGCG-functionalized radioactive gold nanoparticles were shown by Shukla et al. [197] to be able to bypass the vascular and interstitial barriers and deliver medications directly to prostate cancers. The employed gold isotope-98 nanoparticles bind specifically to Laminin67R receptors, which are over-expressed in prostate tumor cells, by exploiting the redox chemistry of a phytochemical that targets prostate tumors. EGCG gold nanoparticles were similarly effective in a mouse melanoma model, as reported by Chen et al. [198] by mitochondrial pathway-mediated apoptosis.

Resveratrol-loaded gold nanoparticles were studied for their potential to combat cancer. After being exposed to an 808 nm laser, gold resveratrol hollow nanoparticles were found to stop cell cycles in A375 melanoma cells, resulting in cell apoptosis and cell death [199]. Research was conducted using gold nanoparticles coated in resveratrol for the treatment of breast cancer cells [200]. Matrix metalloproteinase (MMP)-9 and cyclooxygenase (COX)-2 enzymatic activity and expression were both inhibited by the nanoparticles after being stimulated by thromboxane (TPA). These nanoparticles inhibited phosphoinositide 3-kinase/Akt (PI3K/Akt) and extracellular signal-regulated kinase (ERK)1/2 signaling and blocked the nuclear translocation and transcriptional activation of nuclear transcription factor-kB (NF-kB) and activator protein-1 (AP-1) in response to TPA [200]. After an intravenous injection to mice with colon malignancies, gold nanoparticles coated with technetium-99 m were reported to significantly internalize cancer cells and target colon adenocarcinoma [201]. Another study found that when applied to Hepg2 tumor cells, resveratrol-coated gold nanoparticles significantly inhibited tumor development and decreased VEGF expression [202]. Gold nanoparticles of resveratrol coupled with gum arabic were shown to have anti-angiogenesis characteristics and to undergo efficient endocytosis across breast, prostate, and pancreatic tumor cells [203].

Apigenin glycoside gold and silver quasi-spherical nanoparticles have been reported for use in the hyperthermia of cancer cells and IR-absorbing optical coatings [204]. Epicatechin and theaflavin silver nanoparticles have been shown to have potential efficacy in the human epidermoid larynx carcinoma (Hep-2) cell line through capcase 3 activation and subsequent cell death [205]. Genistein-coated gold nanoparticles with a particle size of 64.6 nm have been shown to have anticancer action against human epithelial lung carcinoma and human melanoma cells [206]. Anticancer medication delivery and bioimaging uses of gold nanoparticles conjugated with kaempferol have been studied [207]. According to the observations, the combination was able to selectively attack cancer cells in the lungs while having minimal effect on healthy tissue.

Quercetin nickel oxide nanoparticles coupled with folic acid as a ligand demonstrated significant effectiveness against the MDA-MB-231 breast cancer cell line and regulated drug release at low pH [208]. Another study found that quercetin absorption was enhanced in SMMC-7721 cells by nickel oxide nanoparticles, leading to a decrease in cancer cell growth [209]. ZnO nanoparticles have been demonstrated to be effective in delivering flavonoids to tumor cells and generating ROS in cancer cells [210]. In addition, ZnO nanoparticles can generate cytokines such as IFN-γ, TNF-α, IL-2, and IL-12, all of which can be used to control the tumor microenvironment [211]. Apoptosis was triggered in human breast cancer cells (MCF-7) by quercetin-conjugated ZnO nanoparticles via an increase in oxidative stress and mitochondrial damage [212]. Similar results were seen by Kundu et al. [213], who found that phenylboronic acid-conjugated ZnO nanoparticles produced curcumin in a pH 5 environment, leading to apoptotic cell death in MCF-7 cells. For naringenin, quercetin, and curcumin, l-histidine-conjugated chitosan with embedded ZnO nanoparticles was produced. These nanoparticles improved their anticancer efficacy against A431 cells [214] after being released in an acidic pH 5 environment. The curcumin ZnO nanoparticles were non-toxic to healthy cells but highly hazardous to rhabdomyosarcoma cell lines [215]. Cell imaging properties and good apoptotic activity in MCF-7 cancer cells were also seen in PEG-beta-cyclodextrin-functionalized ZnO-curcumin nanoparticles [216].

Epithelial-mesenchymal transition (EMT) has recently been recognized as an important regulator of cell invasion and metastasis in cancer [217]. The EMT process contributes to chemoresistance in a number of ways, including the acquisition of migratory/invasive capacities and the creation of cancer stem cells (CSCs). Despite the fact that EMT is a promising therapeutic target for cancer treatment, its use in the clinic is currently restricted for a number of reasons, including heterogeneity in tumor stages, target specificity at the molecular and cellular levels, and effective drug delivery. In order to address the issue, various nanomaterials may be employed to inhibit EMT induction, hence delivering cutting-edge therapeutic options for the treatment of various malignancies. In this regard, nanoparticles made of gold and metal oxides have demonstrated impressive powers in suppressing EMT [218].

The low toxicity, biocompatibility, and stability of gold nanoparticles (AuNPs) have made them widely used as drug delivery methods in biomedicine [218]. Chemotherapy medicines, nucleic acids, and proteins are just a few examples of the bioactive compounds that can be attached to the surface of AuNPs. In in vitro and in vivo models of pancreatic and ovarian cancer, it has been revealed that unaltered AuNPs suppress cell proliferation and metastasis by converting cancer cells from a mesenchymal to an epithelial phenotype [219,220,221]. By inhibiting the activation of the Akt, NF-kB, and MAPK signaling axis, which is essential for EMT, stemness, and drug resistance [219,220,221], the expression of critical stem cell markers and EMT-related markers is reduced.

Gallic acid (GA) is a powerful antioxidant found in plants and many fruits, and it is helpful in suppressing metastasis in glioma [222], gastric [223], and prostate cancer cells [224]. According to certain studies, GA is effective against EMT-related indicators and could be used as a therapeutic drug to treat pulmonary fibrosis in mice [225]. Silver nanoparticles (Ag-NPs) have been shown to be very therapeutic against a wide variety of cancer cells [226] due to their ability to modulate autophagy and serve as cytotoxic agents without any additional help from other agents. Moreover, it has been shown that these nanomaterials can act as nanocarriers to carry therapeutic compounds, such as GA, hindering EMT and so lowering the metastatic potential of A459 lung cancer cells [227].

EGCG is a powerful antioxidant, anti-inflammatory, and EMT inhibitor [228,229,230]. Physiological conditions reduce EGCG’s stability, bioavailability, and metabolic conversion, despite its potent anticancer effects [231,232]. A novel coating membrane, in which EGCG is coordinated with ferric ions to create epigallocatechin gallate/iron nanocomplexes (EIN) [233], has been reported as a potential solution to these problems. Mesoporous silica nanoparticles (MSN) and PEG-PLA micelles (Mic) were each coated with EIN to create MSN@EIN and Mic@EIN, respectively. Both MSN@EIN and Mic@EIN showed excellent biocompatibility, low cytotoxicity, and enhanced intracellular drug concentration upon delivery in breast cancer cells, all of which contributed to the suppression of EMT. These nanostructures inhibited cancer cell migration and drug resistance in addition to killing EMT-type cancer cells in in vivo studies [233]. EGCG-coated MSNs decreased EMT and tumor metastasis when administered to in vivo models of breast cancer [233].

Quercetin has anticancer characteristics since it has been shown to influence tumor cell EMT, proliferation, survival, and differentiation [234,235,236]. Quercetin’s weak solubility in water, however, restricts its use as a medicinal agent. A drug delivery method for quercetin (AuNPs-Quercetin-5) based on gold nanoparticles has been developed and used against breast cancer cells in order to increase quercetin’s efficacy. In MCF-7 and MDA-MB-231 breast cancer cell lines, it was discovered that these nano-conjugates inhibit EMT, which in turn reduces angiogenesis, tumor growth, and metastasis. Intriguingly, 7,12-dimethylbenz(a)anthracene (DMBA)-induced breast cancer in Sprague-Dawley rats was treated with these nanoparticles, and the tumors disappeared [237]. Other strategies have taken advantage of liposomes, which have a number of benefits as nanocarriers, offer great biocompatibility, and enhance the solubility, stability, and pharmacokinetic properties of medications [238]. Regarding this, it has been noted that esophageal cancer stem cells treated with nanoliposomal quercetin displayed reduced EMT and the regulation of numerous EMT-related proteins, including HDAC1 and E-cadherin [239]. The mesenchymal cell’s transition to an epithelial phenotype indicates that these nanoparticles have the potential to treat cancer.

Curcumin has been widely used in conjunction with conventional chemotherapeutics to boost their anticancer activity [240]. Furthermore, recent studies indicated that curcumin inhibits EMT and metastasis in a variety of tumor cells [241,242,243]. Nevertheless, curcumin’s medicinal efficiency is hampered by its poor bioavailability and absorption, quick metabolism, and rapid systemic elimination [244]. This means that particular nanoparticles can be used to boost its bioavailability and pharmacokinetics, allowing for better therapeutic efficacy, internalization, and tumor targeting [244]. Recent research suggests that selenium nanoparticles (Se-NPs) may be effective against prostate, liver, cervical, and breast cancer cells [245,246,247]. For example, curcumin-loaded selenium nanoparticles (Se-Cu-NPs) were found to suppress EMT and reduce inflammation, metastasis, and chemoresistance in colon cancer, whether used alone or in combination with doxorubicin (DOX). This happened as a result of modifications to essential EMT-TFs such as Snail and NF-kB [248,249]. Se-Cu-NPs decreased tumor mass and increased the mean survival time of Ehrlich’s ascites carcinoma (EAC)-bearing mice [248,249].

Nanoforumaltions based on flavonoids are a viable technique for the development of novel, effective anticancer medicines that may supplement current therapies. As a result of their ability to regulate EMT, metal oxide nanostructures show great therapeutic promise for the management of cancer development and metastasis. The benefits of the reported nanostructures should not overshadow the fact that the use of these nanoformulations may exhibit some harm. Modulation of the EMT process may be responsible for both the toxicity and the therapeutic impact [250]. Nonetheless, more research is required to fully explore the potential of flavonoids as a crucial source of anticancer drugs.

### 3.10. Flavonoids in Clinical Trials

A number of trials have been reported during the last several years, given the increasing knowledge of the potential of flavonoids to act as novel cancer therapeutics. Clinical findings from a study conducted by Xiaoling et al. [251] suggest that patients undergoing radiation therapy for esophageal cancer can be successfully treated for acute radiation-induced esophagitis (ARIE) by orally ingesting an EGCG solution. Although EGCG does not interfere with the effectiveness of radiotherapy, it may be a protective strategy against various risks associated with radiation therapy [251].

Nagi et al. [252] discovered that daily intake of a standardized catechin mixture containing EGCG, 200 mg BID for one year, accumulated in plasma and was well tolerated but did not reduce the likelihood of a subsequent prostate cancer diagnosis in men with baseline high-grade prostatic intraepithelial neoplasia (HGPIN) and atypical small acinar proliferation (ASAP). The study [252] found that if appropriate sampling is done at the start, the likelihood of prostate cancer on biopsy within one year of an HGPIN diagnosis is only about 20%. Furthermore, the relatively low one-year rate of prostate cancer seen in men with ASAP in this trial shows that previous reports may have overstated the underlying risk of cancer in that cohort.

In a phase 2 trial for patients with chronic lymphocytic leukemia (CLL), Tait et al. [253] found that once-daily oral administration of EGCG in the Polyphenon E formulation was safe and well tolerated. The majority of patients experienced long-lasting reductions in their absolute lymphocyte count (ALC) and/or lymphadenopathy. The development of more bioavailable oral EGCG formulations is also underway, and these may prove to be more beneficial.

The purpose of another clinical experiment was to evaluate the effects of fisetin supplementation on the inflammatory status and matrix metalloproteinase (MMP) levels among a group of individuals [254]. In a double-blind, randomized, placebo-controlled clinical trial, 37 CRC (colorectal cancer) patients receiving chemotherapy were randomly assigned to receive 100 mg of fisetin (n = 18) or a placebo (n = 19) for seven weeks. The supplementation began one week before treatment and lasted through the end of the second cycle of chemotherapy. Before and after the intervention, plasma levels of interleukin (IL)-8, IL-10, high-sensitivity C-reactive protein (hs-CRP), MMP-7, and MMP-9 were evaluated using ELISA. In the fisetin group, plasma levels of IL-8 and hs-CRP decreased considerably. In addition, supplementation with fisetin decreased MMP-7 levels. However, only IL-8 concentrations in the fisetin group changed significantly compared to the placebo group. Some variations in metabolic factor levels were not statistically significant. According to the data, fisetin could improve the inflammatory status of CRC patients, indicating it as a novel supplementary anticancer drug for these patients and necessitating additional research [254].

The goal of another clinical trial was to see how short-term supplementation with the active components in green tea affected serum biomarkers in patients with prostate cancer [255]. Serum was collected before the medication research began and on the day of the prostatectomy. ELISA was used to examine serum biomarkers such as hepatocyte growth factor (HGF), vascular endothelial growth factor (VEGF), insulin-like growth factor (IGF)-I, IGF binding protein-3 (IGFBP-3), and prostate-specific antigen (PSA). After a brief treatment with EGCG (Polyphenon E), males with prostate cancer had significantly lower serum levels of PSA, HGF, IGF-I, IGFBP-3, and VEGF, with no increase in liver enzymes. In vitro studies showing that EGCG suppresses HGF and VEGF synthesis in cancer-associated fibroblasts supported the decrease in serum HGF and VEGF. These findings suggest that larger, placebo-controlled clinical trials should be conducted to confirm Polyphenon E’s potential significance in the treatment or prevention of prostate cancer. The effects of varying doses, long-term administration, and drug combinations remain to be evaluated [255].

It is believed that sarcoidosis, an inflammatory illness, may have its origins in oxidative stress and insufficient antioxidant levels. Quercetin affects oxidative stress and inflammatory indicators in sarcoidosis [256]. Two groups of age- and gender-matched patients with sarcoidosis who were not treated participated in a double-blind intervention study. Twelve participants received four 500-mg doses of quercetin over the course of 24 h, while six participants received a placebo. Plasma malondialdehyde levels were employed as an indicator of oxidative damage, whereas TNFα/IL-10 and IL-8/IL-10 plasma ratios were used to assess inflammation. The whole plasma antioxidant capacity was raised, suggesting that treatment with quercetin enhanced antioxidant defense. Blood indicators of oxidative stress and inflammation were also decreased in sarcoidosis patients who took quercetin supplements. When oxidative stress and inflammatory markers were higher to begin with, quercetin supplementation appeared to have more of an impact [256].

A small, randomized, double-blind, split-face experiment involving a cream containing 2.5% *w*/*w* EGCG was carried out [257]. Four healthy volunteers with considerable facial erythema and telangiectasia applied EGCG cream to one side of their faces twice daily for six weeks while a vehicle control cream was applied to the other side. Six weeks later, biopsies were collected from EGCG-treated and control locations. VEGF and HIF-1α were quantified using immunohistochemistry. HIF-1α expression was lowered at EGCG-treated sites, with 28.4% of the epidermis staining positively at vehicle-treated sites versus 13.8% at EGCG-treated sites. Similar reductions in VEGF expression were observed in skin treated with EGCG and vehicle control (6.7% and 11.0%, respectively). Topical EGCG therapies affect HIF-1α induction and VEGF expression and may act as a possible drug for telangiectasia prophylaxis [257].

Several other ongoing or completed clinical investigations are looking into the potential use of flavonoids in cancer therapy (Table 3). In the end, those who used these chemicals as an adjunct therapy for cancer treatment or prevention saw encouraging results. The findings summarized indicate that there is still a need for studies to cover multiple gaps in this area. These studies should also take into consideration the various aspects, such as individual response, that could alter the bioavailability and bioactivity of polyphenols [258]. In this situation, databases such as the ClinicalTrials.gov (last accessed on 12 March 2023) registry can play an important role in tracking already active research by keeping an eye on registered intervention studies and reporting any relevant findings.

## 4. Conclusions

Flavonoids are a group of phytochemicals present in a wide variety of plant-based foods, including fruits, vegetables, and beverages such as cocoa, coffee, and tea. It is recommended that a wide range of flavonoids be consumed daily to maintain excellent health and reduce the risk of various life-threatening disorders, including cancer. Flavonoids have been shown to have therapeutic effects in the vast majority of preclinical and clinical studies. In order to develop novel therapeutic medications for a wide range of life-threatening disorders, such as cancer, additional study is certainly needed to elucidate the structures of more flavonoids and investigate their therapeutical potential.

## Figures and Tables

**Table 1:** Flavonoid classes present in diet.

Classes	Representative Flavonoids	Food Sources	References
Flavanol	Catechin, epicatechin, epigallocatechin-3-gallate	Tea	[27,28]
Flavanone	Naringin, naringenin, herperidin	Citrus fruits, oranges, grapefruits, lemons	[29]
Anthocyanins	Cyanidin, peonidin	Blackberry, blueberry, cherry, strawberry	[30]
Flavones	Chrysin, apigenin, luteolin	Celery, green peppers, parsley, peppermint	[31]
Flavonols	Kaempferol, quercetin, myricetin, rutin	Blueberries, apple, cabbage, broccoli, cherries, garlic, onion, tea, red wine	[32]
Isoflavonoids	Daidzein, genistein, glycitein	Legumes, soy	[33]
Tannins (condensed form)	Proanthocyanidins	Cocoa beans, apples, red wine	[34,35]
Deoxyanthocyanidins	Apigeninidin, luteolinidin	Sorghum, purple corn	[36]

**Table 2:** Flavonoid content of various foodstuffs and beverages.

Source of Flavonoid	Total Flavonoid Content (mg/100 g of Food/or Drink)	Major Flavonoid Subclass Present
Black elderberry	1358.66	Anthocyanin
Black chokeberry	1012.98	Anthocyanin
Blackcurrant	608.43	Anthocyanin
Cocoa powder	511.62	Flavan-3-ols
Soybean roasted	253.11	Isoflavonoids
Chocolate dark	237.36	Flavan-3-ols
Blackberry	203.33	Anthocyanin
Broad bean pod	189.54	Flavan-3-ols
Sweet cherry	185.05	Anthocyanin
Black olive	159.83	Anthocyanin
Soy tempeh	147.74	Isoflavonoids
Red onion	131.51	Flavonols
Spinach	119.27	Flavonols
Shallot	112.22	Flavonols
Plum	101.67	Anthocyanin
American cranberry	93.73	Anthocyanin
Black tea	83.35	Flavonols
Aestivalis grape	81.44	Anthocyanin
Green tea	77.44	Flavonols
Common wheat, whole grain flour	77.4	Flavones
Apple	56.35	Flavan-3-ols
Apple juice	54.99	Flavonols
Broad bean seed whole	49.37	Flavan-3-ols
Orange juice	48.02	Flavanones
Grapefruit juice	47.12	Flavanones
Lemon juice	37.43	Flavanones
Buckwheat, whole grain flour	37.04	Flavonols
Barley, whole grain flour	35.2	Flavan-3-ols
Plum juice	30.55	Flavonols
Broccoli	27.8	Flavonols
Red lettuce	22.78	Flavonols
Pistachio	7.193	Flavan-3-ols

**Table 3:** Flavonoids in anticancer clinical studies.

Flavonoid	Cancer Type	Participant Count	FDA Certification Status	Trial Stage	References
Daidzein	Prostate Cancer	43	Phase II	Finished	[259]
Genistein	Prostate Cancer	24	Phase II	On hold	[259]
	Bladder Cancer	60	Phase II	Ongoing	[260]
	Colon and Rectal Cancer	13	Phase II	Finished	[261]
	Non-small Cell Lung Cancer	21	Phase II	Ongoing	[262]
Quercetin	Prostate Cancer	31	Phase I	Ongoing	[259]
	Squamous Cell Carcinoma	55	Phase II	Ongoing	[259]
Apigenin	Colorectal Cancer	382	Phase II	On hold	[259]
Hesperidin	Breast Cancer	40	N/A	Finished	[263]
Catechins	Prostate Cancer	50	Phase I	Finished	[264]
	Breast Cancer	1075	Phase II	Finished	[265]
	Lung Cancer	53	Phase II	Finished	[259]
	Esophageal Cancer	55	Phase I	Finished	[259]
	Cervical Cancer	98	Phase II	Finished	[266]
Cyanidin	Myelodysplastic Syndrome/Myeloproliferative Neoplasm	21	Phase II	Ongoing	[259]
	Oral Cancer	58	N/A	Suspended	[259]
